# Pulmonary embolism following urological non-oncological surgery: The clinical features, management, and long-term follow-up outcome from a tertiary hospital of China

**DOI:** 10.3389/fsurg.2022.930968

**Published:** 2022-09-05

**Authors:** Ziqiang Wu, Xuesong Liu, Quan Zhu, Haozhen Li, Kaixuan Li, Guilin Wang, Zhengyan Tang, Zhao Wang

**Affiliations:** ^1^ Department of Urology, Xiangya Hospital, Central South University, Changsha, China; ^2^ Teaching and Research Section of Clinical Nursing, Xiangya Hospital of Central South University, Changsha, China; ^3^ Provincial Laboratory for Diagnosis and Treatment of Genitourinary System Disease, Changsha, China; ^4^ National Clinical Research Center for Geriatric Disorders, Xiangya Hospital, Central South University, Changsha, China

**Keywords:** pulmonary embolism (PE), urology, non-oncological inpatients, treatment, prognosis

## Abstract

**Objective:**

To evaluate the clinical features, treatment, and outcomes of pulmonary embolism (PE) after urological non-oncological surgery in a tertiary hospital of China.

**Methods:**

A total of eight patients who suffered from PE after urological non-oncological surgery from 2016 to 2019 were recruited to the study. Clinical data such as symptoms, vital signs, electrocardiogram, echocardiography, and computed tomographic pulmonary arteriography (CTPA) were reviewed. In addition, the management and long-term follow-up outcome of PE were reported. Pulmonary Embolism Quality of Life (PEmb-QoL) questionnaire was applied for five patients to evaluate health-related quality of life after PE.

**Results:**

All patients survived during their hospitalization, and five patients were contacted during follow-up. Five of the eight patients were anticoagulated regularly until the re-examination results of CTPA and lower extremities ultrasound were normal. The period of anticoagulant sustained at least one month for each patient. The long-term follow-up outcomes showed that PE had little impact on the patients' quality of life.

**Conclusions:**

The study demonstrated that the prognosis of PE patients was not as terrible as feared when treated immediately in the ward. Early diagnosis and treatment of PE is vital for prognosis. However, further verifications based on the results of large studies are still needed.

## Introduction

Pulmonary embolism (PE), representing a leading cause of perioperative morbidity and mortality, is a serious non-surgical complication following major abdominal and pelvic surgeries (1). The incidence of PE varied between 1% and 10% before the routine use of heparin for preventing venous thromboembolism (VTE) after pelvic surgery (2). The fatality of PE in hospitalized patients was 8.7% in China after the National Cooperative Project for the Prevention and Treatment of Venous Thromboembolism (NCPPT) was conducted (3). In the United States, PE was the third cause of cardiovascular death (4). In urology, the risks of VTE and bleeding in the absence of prophylaxis varied widely in different urological procedures (5). The current literature mainly focused on urological oncological patients (6–8). Nevertheless, the volume of urological non-oncological surgery worldwide is large. Those patients undergoing urological non-oncological surgery are also at risk of PE.

At present, both the American College of Chest Physicians (ACCP) and National Institute for Health and Care Excellence (NICE) recommends extending pharmacological VTE prophylaxis to 4 weeks for high-risk patients undergoing abdominal or pelvic cancer surgery (9, 10). As for patients undergoing urological non-oncological surgery, high-level journal literature has suggested that they do not require regular prophylaxis especially for transurethral resection of the prostate (TURP) (11, 12). However, the evidence that supports the conclusion of the journal literature is of low quality. So, in this study, there was a paucity of data on patients suffering from PE after the performance of the urological non-oncological procedure.

Despite the fact that PE is an uncommon consequence following urological non-oncological surgeries, early detection and treatment are critical in dealing with this possible fatal event. Based on these facts, the clinical features, treatment, and long-term outcome of PE after urological non-oncological surgery in a tertiary hospital are reported in this article.

## Methods

### Patients and clinical data

A total of 8 (0.15%) patients diagnosed with PE out of 5,338 patients in the non-oncological units of the department of urology were included in the study between December 2016 and December 2019. Their clinical data were collected retrospectively, and these were symptoms, vital signs, electrocardiogram, echocardiography, computed tomographic pulmonary arteriography (CTPA), and so on. The relative data were collected through the electronic medical recording system in our hospital. It is worth noting that we collected the highest score of Caprini RAM and the highest D-dimer value of each patient with PE.

### The standard diagnosis and management protocols of PE

The diagnosis of PE was established by the VTE group in our institution according to either CTPA, echocardiography, or suspicious symptoms of PE, such as dyspnea, palpitation, and/or pleuritic chest pain developed after surgery. If PE was suspected, the treatment regimen was determined immediately by consulting the VTE group in our institution. If there were no complications, such as severe bleeding, anticoagulant medication was generally started and continued until the symptoms of PE subsided. Meanwhile, vital signs, liquid intake and output, hemoglobin, and coagulation function were monitored routinely. In general, following the recommendations of VTE experts, low-molecular-weight heparin (LMWH) was subcutaneously injected during hospitalization and rivaroxaban was given orally for one month. The patient will then be suggested to return to the hospital so that the surgeon may assess the situation and determine whether anticoagulation remains necessary.

### Follow-up

The patients were contacted by phone during follow-up. The follow-up time was about 15–37 months after surgery. Information related to symptoms, re-examination findings, and PE's effects on quality of life was comprehensively gathered from the patients. The Pulmonary Embolism Quality of Life (PEmb-QoL) Questionnaire, which was recently developed by Cohn (13), was applied to evaluate health-related quality of life after PE. The questionnaire was completed through telephone follow-up.

### Exclusion criteria

The exclusion criteria of the study were as follows: (1) less than 18 years old, (2) postoperative pathological examination revealing malignancies, and (3) incomplete record.

### Statistical analysis

Categorical variables were presented as counts and percentages (%). Quantitative variables were presented as median and interquartile range (IQR). All statistical analyses were performed by using the software package of IBM SPSS Statistics for Windows, version 22.0 (IBM Corp, Armonk, NY, USA). Only descriptive analyses were performed due to the small number of cases.

## Results

### Baseline characteristics of the enrolled patients

The demographics and perioperative data for the eight enrolled patients are outlined in Table 1. Two (25%) patients were female (patients #1 and #7). The median age of the patients was 65 (IQR: 51.25–67.50). The median BMI of the patients was 24.50 (IQR: 22.73–28.18). The median ASA score was 2.00 (IQR: 2.00–2.50). Concomitant chronic disease such as hypertension was recorded in three cases (37.5%) (patients #2, #4, and #6 in Table 1). Percutaneous nephrolithotomy (PCNL) was performed on two patients (25%) (patients #1 and #4 in Table 1) due to renal stones; TURP was performed on two patients (25%) (patients #2 and #5 in Table 1) due to benign prostatic hyperplasia (BPH); two (25%) patients received the renal artery embolization surgery because of major bleeding complications after PCNL, which was performed in another hospital (patients #6 and #8 in Table 1); one (12.5%) patient underwent laparoscopic right nephrectomy because of hydronephrosis (patient #3 in Table 1); one (12.5%) patient underwent the double J stent placement surgery due to ureteral stones and sepsis (patient #7 in Table 1). The major bleeding complication that required intervention and additional information are also listed in Table 1. We must mention that two (25%) patients (patients #6 and #8 in Table 1) experienced major bleeding and needed intervention after surgery.

**Table 1:** Baseline characteristics of the enrolled patients.

Pt	Gender	Age	BMI (kg/m^2^)	Concomitant chronic diseases	Main diagnosis of urology	Type of surgery	ASA score	Major bleeding requiring re-intervention	Additional information
1	F	51	22.2	None	Multiple nephrolithiasis and hydronephrosis	Right PCNL	2	None	None
2	M	68	26.3	Hypertension	BPH	TURP	2	None	None
3	M	52	23.9	None	Hydronephrosis	Laparoscopic right nephrectomy	2	None	None
4	M	69	25.1	Hypertension	Nephrolithiasis	Left PCNL	2	None	None
5	M	64	22.9	None	BPH	TURP	2	None	None
6	M	51	N/A	None	Nephrolithiasis	Right renal artery embolization	N/A	Yes	Right PCNL was performed 2 days before RAE
7	F	66	33.8	Hypertension	Ureteral calculus and sepsis	DJ stent placement surgery	4	None	None
8	M	66	N/A	None	Nephrolithiasis	Right renal artery embolization	N/A	Yes	Right PCNL was performed 2 days before RAE

BPH, benign prostatic hyperplasia; BMI, body mass index; PCNL, percutaneous nephrolithotomy; TURP, transurethral resection of the prostate; DJ, double J; RAE, renal artery embolization.

### The symptoms, vital signs, and characteristics related to PE

Symptoms of PE were recorded in all the eight patients. Seven (87.5%) patients (except patient #3 in Table 2) shared the main symptoms of dyspnea or shortness of breath. Only one (12.5%) patient (patient #3) developed the symptoms of syncope, cold limbs, and sweating when he got up to go to the bathroom in the middle of the night, 7 days after undergoing right laparoscopic nephrectomy. The median Caprini score was 5.00 (IQR: 4.25–6.75) and the D-dimer was 1.43 μg/ml (IQR: 0.46–4.16). The median hemoglobin level before the occurrence of PE was 128.00 g/L (IQR: 79.75–139.50). The median time for developing the symptoms of PE was three postoperative days (IQR: 1.25–5.50). The median oxygen saturation was 89.50% (IQR: 69.50%–93.75%). Vital signs such as blood pressure (BP), heart rate, and respiratory rate were recorded when the patients developed suspicious symptoms of PE. One (12.5%) patient had systolic blood pressure less than 90 mmHg (patient #3 in Table 2), five (62.5%) had heart rate over 100 beats per minute (patients #1, #3, #5, #6, and #8 in Table 2), and 4 (50%) had a respiratory rate faster than 25 breaths per minute (patients #1, #6, #7, and #8 in Table 2). Five (62.5%) patients (patients #3, #5, #6, #7, and #8 in Table 2) were transferred to the intensive care unit (ICU) for further examination and treatment. The median time of stay in the ICU was 7 days (IQR: 6.50–14.00).

**Table 2:** The symptoms, vital signs, and characteristics related to PE.

Pt	Main symptoms of PE before diagnosis	Caprini score	D-dimer (μg/ml)	Pre-PE hemoglobin (g/L)	Time to PE (postoperative days)	Systolic blood pressure ≤90 mmHg	Heart rate ≥100 bpm^1^	Respiratory rate ≥25 bpm^2^	Oxygen saturation (%)	Transferred to ICU	Days stay in ICU
1	Shortness of breath, flush on face	4	0.23	135	1	None	Yes	Yes	94	None	N/A
2	Dyspnea, chest distress	5	1.62	140	3	None	None	None	74	None	N/A
3	Syncope, cold limbs, sweating	4	0.54	167	6	Yes	Yes	None	88	Yes	7
4	Shortness of breath, chest distress	5	0.43	138	3	None	None	None	96	None	N/A
5	Dyspnea, chest distress, dizzying, sweating	5	4.69	121	2	None	Yes	None	39	Yes	7
6	Shortness of breath, dyspnea, shivering	7	2.55	68	4	None	Yes	Yes	91	Yes	6
7	Shortness of breath	6	7.35	115	9	None	None	Yes	93	Yes	7
8	Dyspnea, shortness of breathing, chest distress, sweating	7	1.23	68	1	None	Yes	Yes	68	Yes	21

PE, pulmonary embolism; bpm^1^, beats per minute; bpm^2^, breaths per minute; ICU, intensive care unit.

### Main risk factors related to PE

We retrospectively analyzed the main risk factors of the eight patients and found that all of them had a history of surgery within one month before PE (Table 3). The median surgery time was 47.50 (IQR: 40.75–73.75) minutes. Meanwhile, five (62.5%) patients had a history of smoking within one year (patients #2, #3, #4, #6, #8 in Table 3) and three (37.5%) patients had concomitant deep vein thrombosis (DVT) (patients #4, #5, #7 in Table 3). However, other risk factors such as malignant tumor, history of VTE, family history of VTE, and varicose veins were not found in these eight patients (Table 3).

**Table 3:** Main risk factors related to PE.

Pt	Surgery time (min)	Blood transfusion	History of surgery in the past month	Malignant tumor	History of smoking within one year	History of VTE	Family history of VTE	Varicose veins	Had concomitant DVT
1	48	None	Yes	None	None	None	None	None	None
2	55	None	Yes	None	Yes	None	None	None	None
3	80	None	Yes	None	Yes	None	None	None	None
4	135	None	Yes	None	Yes	None	None	None	Yes
5	40	None	Yes	None	None	None	None	None	Yes
6	43	None	Yes	None	Yes	None	None	None	None
7	20	None	Yes	None	None	None	None	None	Yes
8	47	None	Yes	None	Yes	None	None	None	None

PE, pulmonary embolism; VTE, venous thromboembolism; DVT, deep vein thromboses.

### Main auxiliary examination characteristics related to PE

**Figure 1:**
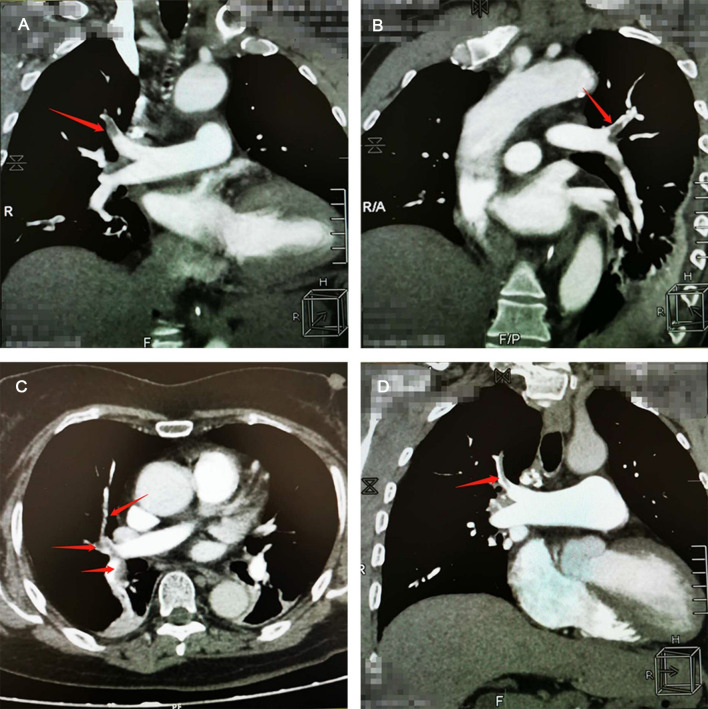
Representative computed tomographic pulmonary arteriography (CTPA) images of pulmonary embolism (PE). (**A**) CTPA shows filling defects in the right upper pulmonary artery (red arrow). (**B**) CTPA shows filling defects in the left upper pulmonary artery (red arrow). (**C**) CTPA shows filling defects in the right upper pulmonary artery, right middle pulmonary artery, and right lower pulmonary artery (red arrow). (**D**) CTPA shows filling defects in the right upper pulmonary artery (red arrow).

**Figure 2:**
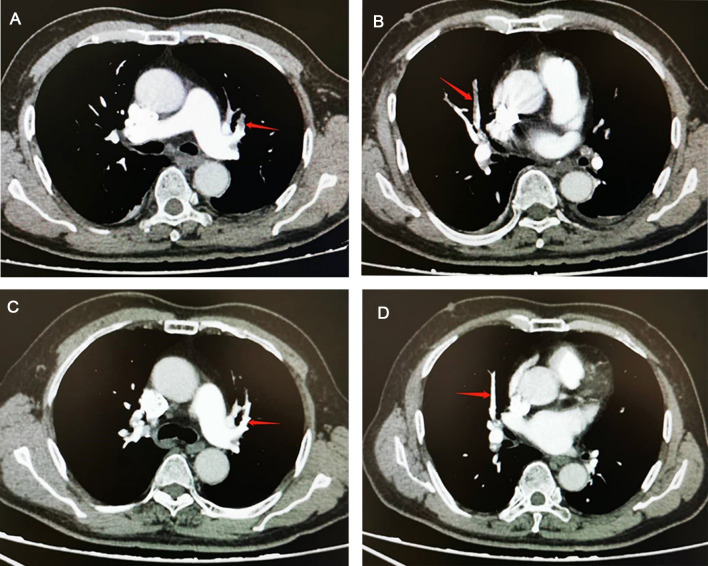
Comparison of computed tomographic pulmonary arteriography (CTPA) images before and after treatment in a pulmonary embolism (PE) patient. (**A**,**B**) CTPA shows filling defects in the left upper pulmonary artery and right upper pulmonary artery (red arrow). (**C**,**D**), CTPA shows that filling defects in the left upper pulmonary artery and right upper pulmonary artery have disappeared after administering anticoagulants for one month (red arrow).

Main auxiliary examination data for the eight patients, such as echocardiography, electrocardiogram, and CTPA, are presented in Table 4. Representative CTPA images of PE are presented in Figure 1 (A, B, patient #5 in Table 4; C, patient #7 in Table 4; D, patient #2 in Table 4). Figure 1A shows filling defects in the right upper pulmonary artery (red arrow). Figure 1B shows filling defects in the left upper pulmonary artery (red arrow). Figure 1C shows filling defects in the right upper pulmonary artery, right middle pulmonary artery, and right lower pulmonary artery (red arrow). Figure 1D shows filling defects in the right upper pulmonary artery (red arrow). A comparison of CTPA images before and after treatment of PE is shown in Figure 2 (patient #4 in Table 4). Figures 2A,B show filling defects in the left upper pulmonary artery and right upper pulmonary artery (red arrow). Figures 2C,D show that the filling defects disappeared after administering anticoagulants for one month (red arrow). Seven (87.5%) patients (except patient #3 in Table 4) were diagnosed with PE by using CTPA. Only one (12.5%) patient (patient #3 in Table 4) was found unsuitable to take CTPA examination and was indirectly diagnosed by echocardiography combining with the symptoms of syncope, cold limbs, sweating, and oxygen desaturation.

**Table 4:** Main auxiliary examination characteristics related to PE.

Pt	Echocardiography	Typical electrocardiogram of PE	CTPA
1	Both ventricles with normal size and function, normal pulmonary arterial pressure	None	Little perfusion absence in the dorsal segment pulmonary artery in the right lower lobe
2	RV filled, pulmonary artery slightly widened, LA/LV slight dilation	None	PE in the apical segment of the right upper lobe、the dorsal segment of the right the lower lobe, and the posterior basal segment of the left lower lobe
3	RA/RV dilation, Inferior vena cava enlarged, pulmonary arterial hypertension	None	N/A
4	N/A	None	Bilateral pulmonary embolism
5	Acute enlargement of RV, pulmonary arterial hypertension	None	Multiple PE in both lungs
6	N/A	N/A	PE in the lingual segment of the left upper lobe and the lateral basal segment of the left lower lobe
7	N/A	N/A	PE in the right pulmonary artery trunk, the right upper pulmonary artery, the right middle pulmonary artery, the right lower pulmonary, and its basal branches.
8	Pulmonary arterial hypertension, enlargement of RV outflow tract, LA dilation	Yes	Filling defect in the right upper pulmonary artery

PE, pulmonary embolism; RA, right atrium; RV, right ventricle; LA, left atrium; LV, left ventricle; CTPA, computed tomographic pulmonary arteriography.

### Treatment and complications of PE

Anticoagulation was initiated in all eight patients with low-molecular-weight heparin (LMWH), unfractionated heparin (UFH), enoxaparin, or dalteparin. Thrombolytic therapy was applied in one (12.5%) patient (patient #3 in Table 5) with recombinant tissue plasminogen activator (rt-PA) because of hemodynamic instability. However, anticoagulants were not continued in two (25%) patients (patients #4 and #8 in Table 5) because of gross hematuria and hemorrhage in the surgical site after 3 days and 8 days of giving anticoagulants, respectively. Six (75%) patients (except patients #2 and #8 in Table 5) were using mechanical prophylaxis like intermittent pneumatic compression (IPC) before diagnosis of PE. Anticoagulant complications such as small hematoma at the injection site, intracranial hemorrhage, low platelet count, and subcutaneous bruises were not detected in these eight patients (Table 5).

**Table 5:** The treatment regimens and complications of PE.

Pt	Anticoagulant therapy during stay in hospital	Phromboprophylaxis before PE	Small hematoma at injection site	Intracranial hemorrhage	Low platelet count	Subcutaneous bruises	Anticoagulation needs to be stopped due to bleeding complications	Hemorrhage in the surgical site
1	Rivaroxaban, 2 days	IPC	None	None	None	None	None	None
2	LMWH, 3 days; LMWH, 4 days	None	None	None	None	None	None	None
3	rt-PA, 2 h; dalteparin, 8 days; UFH, 6 days	IPC	None	None	None	None	None	None
4	LMWH, 3 days	IPC	None	None	None	None	Yes	Yes
5	Enoxaparin, 8 days; enoxaparin, 9 days; rivaroxaban, 2 days	IPC	None	None	None	None	None	None
6	LMWH, 10 days.	IPC	None	None	None	None	None	None
7	Enoxaparin, 6 days; urokinase, 12 days	IPC	None	None	None	None	None	None
8	UFH, 8 days; LMWH, once	None	None	None	None	None	Yes	Yes

LMWH, low-molecular-weight heparin; UFH, unfractionated heparin; IPC, intermittent pneumatic compression; rt-PA, recombinant tissue plasminogen activator.

### Follow-up data of PE patients

Follow-up data were obtained for five (62.5%) patients (patients #1, #2, #4, #5, and #7 in Table 6). The median follow-up time was 28 (IQR: 16.50–36.00) months after surgery. Four (50%) patients (patients #1, #2, #4, and #7 in Table 6) secured 1 point for the six dimensions (frequency of complaints, FO; limitations in activities of daily living, AD; work-related problems, WR; social limitations, SL; intensity of complains, IO; emotional complaints, EC) of PEmb-QoL. One (12.5%) patient secured 1.1 points for the dimensions of FO and AD in the PEmb-QoL questionnaire and 1 point for the remaining four parameters. For all dimensions, a score of 1 point means no complications. Just one (12.5%) patient (patient #5 in Table 6) complained of chest distress incidentally when running, whereas the other four (50%) patients had no symptoms of PE. Two (25%) patients (patients #1 and #4 in Table 6) were anticoagulated for 3 months, one (12.5%) (patient #5 in Table 6) was anticoagulated for one month, and one (12.5%) (patient #2 in Table 6) was anticoagulated for 6 months by administering rivaroxaban, respectively. Only one (12.5%) patient (patient #7 in Table 6) has been anticoagulating irregularly with warfarin for 35 months. All the five (62.5%) patients were re-examined by using CTPA and lower extremities ultrasound. The reviewed results of CTPA and lower extremities ultrasound for these patients were normal (Table 6).

**Table 6:** Follow-up data.

Pt	PEmb-Qol score						Symptoms of PE	Information on taking medicine at home	Re-examination results of CTPA	Re-examination results of ultrasound	Follow-up time (months)
	FO	AD	WR	SL	IO	EC					
1	1	1	1	1	1	1	None	Rivaroxaban, 3 months	Normal	Normal	37
2	1	1	1	1	1	1	None	Rivaroxaban, 6 months	Normal	Normal	18
3	N/A	N/A	N/A	N/A	N/A	N/A	N/A	N/A	N/A	N/A	N/A
4	1	1	1	1	1	1	None	Rivaroxaban, 3 months	Normal	Normal	28
5	1.1	1.1	1	1	1	1	Chest distress sometimes when running	Rivaroxaban, 1 months	Normal	Normal	15
6	N/A	N/A	N/A	N/A	N/A	N/A	N/A	N/A	N/A	N/A	N/A
7	1	1	1	1	1	1	None	Warfarin, 35 months	Normal	Normal	35
8	N/A	N/A	N/A	N/A	N/A	N/A	N/A	N/A	N/A	N/A	N/A

FO, frequency of complaints; AD, ADL (ADL, activities of daily living) limitations; WR, work-related problems; SL, social limitations; IO, intensity of complaints; EC, emotional complaints; PE, pulmonary embolism; CTPA, computed tomographic pulmonary arteriography.

## Discussion

In this study, all the PE patients survived up to discharge. Also, we found that the prognosis was not as bad as we expected if the PE was diagnosed early and treated as soon as possible. According to the current literature, the rate of PE in patients undergoing urological non-oncological surgeries such as TURP and PCNL is low (14, 15), and the guidelines mainly focus on the postoperative VTE of abdominal or pelvic cancer patients (9, 16). Although PE is not common after urological non-oncological surgery, it is fatal if not treated immediately. Therefore, it is crucial to enhance the diagnostic and treatment capabilities of PE early.

Four of the eight PE patients received PCNL. Two of the four patients had complications of major hemorrhage after PCNL in other hospitals and were transferred to our hospital by ambulance for further treatment. Then, renal artery embolization was performed on those two patients. As we all know, PCNL is a well-established treatment method for patients with nephrolithiasis (17–19). What is more, PE after PCNL is an uncommon complication (15). Nevertheless, cases of patients who died of PE after PCNL have also been reported (20, 21). Shin et al. reported that one patient died of PE in their series of 698 patients who underwent PCNL (20), and Hentschel et al. reported one death in their series of 158 cases (21). Recently, Paparidis et al. reported a case of PE on the first postoperative day and who survived after timely treatment (22). PE is a rare but life-threatening complication that occurs after PCNL.

Seven of the eight patients in our study presented with some specific symptoms related to the respiratory system, such as dyspnea and shortness of breath accompanied by oxygen desaturation. One patient presented with general symptoms such as syncope, cold limbs, and sweating. This was consistent with that of previous studies (23). This result suggests that specific or general manifestation plays an important role in the early diagnosis of PE. Six of the eight patients were classified as high-risk level of VTE according to the Caprini risk assessment model (RAM), which was recommended by the ACCP for evaluating the risk of VTE for non-orthopedic surgical patients (9). D-dimer levels in six patients were higher than 0.5 µg/ml and those in five of the six patients were even higher than 1 µg/ml. This indicates that we should pay special attention to high-risk VTE patients with increased D-dimer levels during the perioperative period.

When we searched the main risk factors of PE, we found that all of the patients had a history of surgery. Meanwhile, five patients underwent surgery longer than 45 min. Also, five of the eight patients had a history of smoking within 1 year prior to PE. Surgery and smoking are important risk factors of PE that are listed in the guidelines of the European Society of Cardiology (24). Smoking may change the micro environment of the pulmonary vascular and lung tissue. Since surgery may destroy the integrity of the blood vessels, it is also a risk factor of blood clotting. In this research, three of the eight patients were combined with DVT. This was consistent with that of previous studies. Jiménez et al.and Yamaki et al. reported that 51% and 58% of the PE cases had concomitant DVT when suffered from PE (25, 26). We speculate that fibrin can form in the pulmonary vessels without appearing from the periphery. Seven of the eight patients were diagnosed by using CTPA. It is reported that the diagnostic accuracy of CTPA for PE is between 68% and 97% (27) and it is the gold standard for the diagnosis of PE (28). In most of the cases, we have directly seen the filling defect in the pulmonary artery through CTPA. Only one patient was not diagnosed by using CTPA because of severe symptoms of hemodynamic instability, while his echocardiography revealed a dysfunction of the right ventricle. Two patients developed a hemorrhage in the surgical site, warranting the stopping of anticoagulants. Peris et al. reported that the rate of major bleeding was 10.1% during the course of anticoagulant therapy in cancer patients who experienced incidental PE (29). This suggests that during the course of anticoagulation, it is important to monitor the activated partial thromboplastin time (APTT), prothrombin time (PT), and international normalized ratio (INR) to detect coagulopathy and bleeding early and adjust treatment regimens as soon as possible.

In the follow-up, we found that PE had little influence on the patients' quality of life according to the PEmb-QoL questionnaire. Previous studies have revealed that the PEmb-QoL questionnaire is a reliable instrument to assess the quality of life following PE (30–32). Sun et al. reported that the PEmb-QoL questionnaire is both valid and reliable to measure QoL following PE in Chinese patients (33). So, we speculate that most of the urological non-oncological inpatients suffering from PE have a good prognosis if treated immediately and properly upon the occurrence of PE.

This study is retrospective in nature and is beset with several limitations. First, one of the eight patients was not diagnosed by using CTPA and instead his diagnosis depended on his symptoms, vital signs, and echocardiography. Second, only five patients were contacted and were willing to cooperate with our follow-up. Third, not all the patients were re-examined in our hospital and so we could obtain only the re-examined images of CTPA for three patients. Fourth, the patients' all-cause mortality were not taken into consideration. Therefore, further research studies on these patients are needed. Finally, only eight cases were included in this study. From this point of view, our study was merely to present the clinical manifestations, treatment, and outcomes of PE in urological non-oncological individuals. Therefore, multicenter research studies with large samples are needed.

## Conclusion

The prognosis of inpatients suffering from PE after urological non-oncological surgery was not as terrible as feared when treated immediately in the ward. It can be concluded that early diagnosis and treatment of PE is vital for prognosis. However, further verifications of the results by multicenter studies are still needed.

## Data Availability

The original contributions presented in the study are included in the article/Supplementary Material, further inquiries can be directed to the corresponding author.
